# Supplementary material for: Barriers and facilitators to digital technology application for antimicrobial resistance surveillance: A co-produced qualitative synthesis
Source: PLOS Glob Public Health. 2025 Jul 23;5(7):e0004894. doi: 10.1371/journal.pgph.0004894 (PMC12286375; doi:10.1371/journal.pgph.0004894)
Supplement: S1 Text — (DOCX) [file pgph.0004894.s001.docx]

**Barriers and facilitators to digital technology application for antimicrobial resistance surveillance: a co-produced qualitative synthesis**

**S1. Key Informant Interview Guide**

**Section A: Socio-demographic Characteristics**

1. Gender:
2. Current Position:
3. Speciality:
4. Time (mm/yr) of introduction to data
5. Years of experience with data:
6. Experience level with data (beginner/intermediate/expert)
7. Voluntariness to use the system for capturing data
8. Level of technology use (beginner/intermediate/expert)

**Section B: UTAUT**

1. Do you think the use of a Laboratory Information Management System (LIMS) such as WHONET is necessary?
   1. *Does it enhance your job, and how?*
   2. *How might the use of WHONET be different from other record-keeping processes, such as recording in notebooks?*
   3. *What do you think the data that you capture with WHONET is being used for*
2. How difficult or easy is capturing data from your lab in the required/appropriate format on WHONET?
   1. *What makes it easy or difficult (challenges experienced)?*
   2. *How does WHONET integrate with other LMIS you use in your lab?*
   3. *How much effort is required to capture your lab data fully?*
3. Do you think other labs might be doing better or worse in capturing data on WHONET in the appropriate/required format?
   1. *Have you heard complaints from your colleagues at other sites regarding the challenges they might be facing?*
   2. *What does your line manager think about using WHONET to capture your laboratory data in the appropriate format?*
4. Do you have the necessary skills and support to capture your lab data on WHONET?
   1. *Does your institution offer any training to overcome challenges encountered or make your work easier using LIMS?*
   2. *If you had the power, what structures would you put in place to support capturing data from your laboratory to meet the required standards?*
      1. *It could be structures at your lab, the national coordinating centre, nationally, or anywhere else you could think of.*
