# Supplementary material for: Barriers and facilitators to digital technology application for antimicrobial resistance surveillance: A co-produced qualitative synthesis
Source: PLOS Glob Public Health. 2025 Jul 23;5(7):e0004894. doi: 10.1371/journal.pgph.0004894 (PMC12286375; doi:10.1371/journal.pgph.0004894)
Supplement: S2 Text — (DOCX) [file pgph.0004894.s002.docx]

**S2. PAR Workshop Discussion Guide**

**Section A: Introduction**s

**Section B: Problem Identification**

What other challenges *(aside from those already identified)* do you *(or people at your sentinel)* experience with using a laboratory information management system like WHONET at your sentinel lab?

- Probe any previously identified challenge.
- Does any other sentinel experience this challenge? How?
- How significant is this challenge or other challenges previously identified?

**Section C: Solutions Identification**

What strategies might be put in place to address the challenges you have identified today and those shared previously?

- Does any other sentinel think that this strategy would be viable for tackling the same challenge at their sentinel?
- Which stakeholder might be responsible for implementing this strategy?
