# Supplementary material for: Barriers and facilitators to digital technology application for antimicrobial resistance surveillance: A co-produced qualitative synthesis
Source: PLOS Glob Public Health. 2025 Jul 23;5(7):e0004894. doi: 10.1371/journal.pgph.0004894 (PMC12286375; doi:10.1371/journal.pgph.0004894)
Supplement: Attachment — Submitted filename: 02_response__AMR Surveilance__Plos_GH.doc [file pgph.0004894.s004.doc]

June 2025

Dear Editor and Anonymous Reviewers:

Thank you for another opportunity to revise the manuscript

“Barriers and facilitators to digital technology application for antimicrobial resistance surveillance: a co-produced qualitative synthesis”

for reconsideration for publication in *PLOS Global Health*. We appreciate the time taken to critically review this paper and the constructive feedback and insights from the reviewers. We have carefully considered all the reviewers’ comments and have addressed each of the concerns as outlined below. In addition to these, we noticed during further review that the 23 individuals who participated in the in-depth interviews were from eight sentinel labs. We have now updated Figure 1 and all other instances accordingly. We have also carefully reviewed the manuscript and data to ensure our reporting is accurate.

**Reviewer 1 Comments:**

***Major Comments***

1. Baseline demographics of the nine participating sentinel sites and 23 participants in the in-depth interviews are largely unclear. Table 1 notes that 19 participants were "biomedical scientists," while the text on line 7 of page 9 refers to them as "laboratory scientists." Are they different?

***Response:*** *We have carefully rephrased all references to "laboratory scientists" and "biomedical scientists" and changed them to “laboratory technologists/scientists” for consistency and to reflect the broad range of disciplines. Also, see page 8, Table 1 and page 9, Table 2.*

Additionally, it is stated that "most" perform data capture "voluntarily." It is unclear to readers what the "full-time jobs" of these 23 participants (and 4 data officers) are? Who pays their salaries? Universities as academic staff or Hospitals as laboratory staff (but called scientists / data officers)? How many of them are full-time academic staff whose roles include research, teaching, and publishing manuscripts? How many are full-time laboratory staff hired by university hospitals to perform routine laboratory work, data entry, data analysis, and report submissions? Do the university hospitals or university laboratories hire any non-academic persons to do such tasks? Are any of the full-time academic staff funded by AMR grants or the Fleming Fund, with FTE dedicated to supporting WHO GLASS per contract, to do such tasks? Can this part be clarified?

***Response:*** *The full-time jobs of the 23 participants have now been clarified in the manuscript and included here: “19 of the 23 participants are full-time laboratory technologists/scientists”. None of the 23 participants are full-time academic staff members with research or publishing responsibilities, although three medical doctors who participated in the co-design workshop and feedback session are full-time academic staff whose roles include teaching and research. All the laboratory staff (biomedical laboratory scientists) are hired and paid by the hospital to perform routine laboratory work. Only four of the sentinel labs have so far hired a non-academic person to support the laboratory with data entry. Although almost all the laboratories included in this study (apart from UBTH) receive support from the Fleming Fund, this support does not currently extend, at least to our knowledge, to the funding of a dedicated data scientist responsible for entering AMR surveillance data. Specifically, there is no formal provision within existing Fleming Fund contracts for full-time equivalent (FTE) staff assigned to data entry tasks in support of WHO GLASS reporting.*

2. Although the participants in the in-depth interviews are 'voluntary researchers' and noted that their time supporting 'data capture' is voluntary, it is possible that these nine participating sentinel sites have full-time laboratory personnel tasked with 'data entry,' 'data analysis,' and 'report submission.' Can the availability or lack of full-time laboratory personnel be clarified with proper numerators and denominators?

For example, "XXX out of nine sentinel laboratories have at least one full-time laboratory personnel responsible for 'data analysis' and 'report submission' to the National Coordinating Centre, while the remaining XXX sentinel laboratories do not."

***Response:*** *Participants in the in-depth interviews are laboratory scientists (19) and data officers (4) who are directly involved in inputting laboratory results into the LIMS (either as their primary or volunteer role). We have also clarified in the manuscript that: “Four out of eight sentinel laboratories that participated in the in-depth interview have at least one full-time personnel responsible for data entry and report submission to the National Coordinating Centre, while the remaining four sentinel laboratories do not.” We have also included this information on page 5, LN 12-14 of the clean document*

Although this is a social science study, baseline information is important for readers to understand the context. This is exemplified on page 12, line 20: "Two out of seven labs assessed did not have stock management mechanisms in place." More descriptions using denominators of all labs with data would be helpful to readers and provide more impact to policymakers. (I assume that the remaining two labs do not have data about stock management mechanisms, if not typo. Can this point be clarified in the text?)

***Response:*** *Thank you for your helpful comment. Unfortunately, we could only assess seven of the eight labs that participated in the in-depth interview. The remaining lab was unavailable for the assessment during the interviews. We have also added this clarification in the revised manuscript. Please see page 13, LN 4-5:*

*Two out of seven sentinel labs assessed (one sentinel was not accessible for assessment) did not have stock management mechanisms in place, which disrupted their ability to administer proper patient care and services.*

3. As the key topic of the study is WHONET and LIMS, can the authors describe clearly from the interviews:

- What LIMS (name of the LIMS and companies) are being used daily in the nine study labs?
- Do any labs use MS Excel or MS Access as LIMS?

***Response:*** *The primary LIMS used across the eight (8) sentinels is WHONET. In addition, these labs use other LIMS daily for further data capturing. This includes: SEDRILIMS is used at one site (OAUTHC), RAPID MEDCHART at one site (UBTH), Health-in-a-Box at three sites (AKTH, UCH and UCTH), and REDCap is used occasionally at one site (UDUTH). Five (5) out of the eight (8) sentinel lab - AKTH, FMCJ, UCTH, UDUTH, and UITH use Microsoft Excel either regularly and/or for exporting data to WHONET. Nevertheless, all eight labs routinely use notebooks to record data before transferring to LIMS. We have also included this information on page 10, LNs 28-32, page 11, LNs 1-8 of the clean document*

- Do all nine labs perform double data entry in WHONET and their own LIMS?

***Response:*** *Most of the labs perform double entry in some way (WHONET + their LIMIS, WHONET + Notebook, WHONET + Excel). We have also clarified this in the main manuscript. Please see reference above.*

- Do none of the nine labs use BacLink? Although page 10, lines 15-26, describes some issues about LIMS, Health-in-a-Box, and BacLink, and it is clear that all hospitals use "Health-in-a-Box" as a Hospital Management Information System, it is unclear how many are using LIMS, which LIMS, and BacLink. BacLink could be difficult, but "many" (line 23) is unclear whether none can use it at all in Nigeria.

***Response:*** *Only two of the eight labs (UCTH and UCH) use BacLink alongside WHONET, while the other sentinel laboratories do not. This is either due to challenges in using the tool such as limited technical capacity, compatibility issues with their existing systems or because they have not needed it as they do not have the necessary backlog of data requiring export into WHONET. We have also clarified this in the main manuscript. Please see page 11, LN 12-15.*

- Are there any hospitals performing triple or quadruple data entry (e.g., Health-in-a-Box, LIMS, WHONET, and notebooks)? Can this part be clarified with proper numerators and denominators (by number of hospitals)?

***Response:*** *All eight sentinel laboratories perform at least triple data entry, commonly using WHONET and notebooks alongside one or two other LIMS. Three (3) of these 8 labs (AKTH, UCTH, and UDUTH) perform quadruple data entry, while the remaining 5 perform triple data entry. We have also clarified this above with appropriate reference to sentinels using specific LMIS. Please see page 10, LN 28-32 in the revised manuscript.*

4. I am uncertain about the question, "Have the authors made all data underlying the findings in their manuscript fully available?" Although the authors stated that "All data used for the study are included in the paper," interview guides (including question list and other related documents), individual-level anonymous data/quotes from participants are not provided or deposited in a public repository. The data and quotes related to each theme are not fully available; only few selected quotes are shown in the text. I wonder whether the authors could provide more supporting data as an appendix or to a public repository. This is highlighted by the fact that readers could be unclear whether other points were asked in the interview and what the anonymous answers/quotes to those points were.

***Response:*** *Thank you for your suggestion. We have included a copy of the interviewer guides for both the in-depth interview and the co-design workshop as supplementary materials. We have also included more quotes to reflect the participant views. Unfortunately, we are unable to share full transcripts from the interviews due to ethical reasons (participants may become easily identifiable). We have also updated our data availability section to reflect this. Please see the following sentence on page 20 (LNs 26-31):*

*“Some qualitative quotes derived from the study are included in the paper. Due to the small number of sentinels involved, we are unable to share full transcripts from the in-depth interviews and participatory workshop. Sharing full transcripts publicly may compromise participant anonymity and affect individuals’ professional roles and confidentiality. Nevertheless, anonymised excerpts could be made available upon reasonable request to the corresponding author.”*

***Minor Comments***

1. The terms LIMS and LIS (page 15, lines 2 and 4): Is LIS a typo? Do the authors mean LIMS? If the authors intend to differentiate between LIS and LIMS, can they provide definitions or details about the differences between LIS and LIMS?

***Response:*** *Yes, we meant LIMS and not LIS. We have also reviewed the manuscript and ensured consistency throughout.*

2. Can the authors clarify in *intro* methods and/or discussions that LIMS in this work focuses solely on "microbiology LIMS," which can differ from general LIMS used in clinical laboratories? Did this distinction cause confusion for any participants? Do the laboratories in Nigeria use different general LIMS and microbiology LIMS? If yes, can this be added into the discussion?

***Response:*** *Thank you for your thoughtful suggestion. We have revised the manuscript to clarify that our study focuses solely on microbiology LIMS. This is because the AMR surveillance system in Nigeria only engages the department of medical microbiology and parasitology, not other units within the medical laboratory sciences. Therefore, this distinction did not cause confusion among participants. Furthermore, while sentinel laboratories use different microbiology LIMS, our study did not investigate whether they also use different or the same general LIMS across departments, as our scope was limited to the microbiology and parasitology units. We have further clarified this in the introduction section (please see page 2, LNs 30-32) and methodology (please see page 5, LNs 16):*

*“Despite this, a clear picture of the barriers and facilitators of efficient use of laboratory information management tools and systems (LIMS) in* ***microbiology laboratories*** *is yet to emerge.”*

3. Can the authors describe or discuss whether Health-in-a-Box includes functions for both "general LIMS" and "microbiology LIMS"? Can details obtained from participants during the interviews be provided to readers in the manuscript?

***Response:*** *Health-in-a-Box is a Hospital Information Management System (HIMS) and not a Laboratory Information Management System (LIMS). Although it can be used to capture patient demographics, it is solely used for tracking hospital finances. In addition, Health-in-a-Box has a full-time, dedicated data officer who is employed and paid by the hospital to enter data into the system. This distinction has also been made explicit on page 10, LN 30-32.*

4. The authors frequently use the term "capture data" (e.g., page 9, line 10). Can the authors clarify whether this means "input data," "export data," or "data analysis"? For example, do the interviewees (academic scientists) support the hospital laboratories by inputting data into WHONET, exporting data from LIMS, importing data into WHONET, and analyzing data using WHONET? Or some functions of those? Or do they mean something else by "capturing data"?

***Response:*** *By "capturing data," we mean the complete process of manually inputting laboratory results and relevant metadata into WHONET or any other Laboratory Information Management Systems (LIMS) used by the laboratories. This refers specifically to data entry and does not include data export, import, or analysis functions. We have further clarified this in the tracked manuscript. See page 11, lines 14-15 and page 12, LNs 1-2*

5. Acronym of LIMS (page 16, lines 27 and 28): Should "laboratory information management systems" be abbreviated as LIMS here?

***Response:*** *Yes. "Laboratory Information Management Systems" should be abbreviated as* ***LIMS*** *in this context, and we have revised this accordingly. Please see changes on page 18, LN 5 & page 19, LN31 in the revised manuscript. We have also thoroughly reviewed the manuscript and ensured consistency throughout.*

**Reviewer 2 Comments:**

1. The participation from the National coordinating center is 0 at the interview and participatory co-design workshop stage though their participation in the feedback session is clear. This could be a limitation that prevented their perspective from being included. This must be mentioned as a limitation.

***Response:*** *Thank you for your feedback. The primary focus of the interviews and participatory co-design workshops was to engage stakeholders who were directly involved in using WHONET to capture laboratory data. The NCC, however, is not a direct stakeholder in this context. As a result, their participation was limited to the feedback session to ensure they understood the challenges identified by frontline stakeholders and to gather their reflections on the proposed solution. This sequencing allowed for appropriate role-based involvement without compromising the study’s objectives. Please see changes on page 19, LNs 13-19 in the revised manuscript.*

*“Furthermore, there was limited involvement of the national coordinating centre in in-depth interviews and the participatory co-design workshop. Although their broader system-level perspective may have provided additional insights beyond those captured from frontline stakeholders, we prioritised perspectives from stakeholders who were directly involved in using WHONET to capture laboratory data in these phases. Nevertheless, their participation in the feedback session provides an opportunity to gather their input and reflections on the proposed solution.”*
